# Does the New Rural Pension System Promote Farmland Transfer in the Context of Aging in Rural China: Evidence from the CHARLS

**DOI:** 10.3390/ijerph16193592

**Published:** 2019-09-25

**Authors:** Yahui Wang, Qingyuan Yang, Liangjie Xin, Jingyu Zhang

**Affiliations:** 1 School of Geographical Sciences, Southwest University, Chongqing 400715, China; wangyahui1210@163.com (Y.W.); zhangjy@swu.edu.cn (J.Z.); 2 State Cultivation Base of Eco-Agriculture for Southwest Mountainous Land, Southwest University, Chongqing 400715, China; 3 Institute of Geographic Sciences and Natural Resources Research, Chinese Academy of Sciences, Beijing 100101, China

**Keywords:** new rural pension system, farmland transfer, population aging, panel logit model, panel tobit model, CHARLS

## Abstract

The lack or instability of the pension system for the elderly in rural China has become a paramount obstacle for sustainable land transfer, namely land use right transfer among farmers, in the context of aging. The New Rural Pension System (NRPS), a pilot project that provided basic security for the elderly, was implemented in 10% of counties in 2009 and rapidly promoted nationwide in China. This study evaluates the impact of NRPS on farmland transfer by developing econometric models by employing the China Health and Retirement Longitudinal Study (CHARLS) from 2011 to 2015. The participation rate in NRPS increased from 25.87% in 2011 to 80.85% in 2015, and the participation rate in farmland transfer rose from 11.56% to 24.04%. Everything else being held equal, the probability of farmers who transferred out their land increased by approximately 13% and the land area has been transferred increased by 11.2% due to participation in NRPS, indicating that the NRPS improved the operation efficiency of land rental market. Furthermore, the heterogeneity analysis showed that the probability and area mentioned above had a significant upward trend with the increase of the time and insured amount of participation in NRPS, which reduced dependence on farmland for the elderly and promoted the sustainability of land transfer. The government should further encourage farmers to increase the coverage and insured amount of pension system in the context of aging. Meanwhile, a platform to promote land transfer should be established to provide information about land supply and demand and reduce the transaction cost of land rental market.

## 1. Introduction

Land productivity, namely grain output of cultivated land per unit, in China was 5520 kg in 2010 according to the World Bank; it ranked 16th compared with 128 counties or regions in the world, well above the world average of 3563 kg [1]. However, agricultural labour productivity represented only 47% of the world average, approximately 2% of the average for high-income counties and 1% of that of the United States and Japan [1,2]. Agricultural development in China presents an awkward situation of high grain yield per unit (one long leg) and low labour productivity (one short leg). The root cause of low agricultural labour productivity is that family farms are still small [2,3]. Small farm size has become a crucial bottleneck in agricultural development because it is difficult to reach an appropriate scale [4,5,6].

Recently, considerable literature has shown that farmland transfer, namely land transfer among farmers, is an effective way to realize large-scale agricultural operation and promote the agricultural modernization in rural China [7,8,9,10]. With the continuous rise in non-agricultural wages, the rural labour is flowing to cities on a large scale, and extensive utilization for farmland and land abandonment are increasing [10,11,12,13]. A study by the Chinese Academy of Sciences found that 78% of mountain villages had experienced land abandonment between 2014 and 2015 [13]. To improve the allocation efficiency of land resources, they should be transferred from those who are unwilling to manage farming or have relatively low agricultural productivity to those who are willing to manage farming or have higher agricultural productivity [14]. The Several Opinions on Accelerating Reform and Innovation to Accelerate Agricultural Modernization issued by document No. 1 in 2015 clearly stated the aim of innovating the land transfer modes and developing a variety of forms of operations to appropriate scale. Currently, farmland transfer has been regarded by the government as a paramount way to promote the large-scale operation of agricultural land.

By 2018, the transferred area of farmland with contract rights has reached 26.87 million hm^2^, which was approximately 20% higher than the amount in 2012 [15]. The transferred area accounts for 31% of the total contracted farmland and 25% of farmers participate in land transfer. In recent years, the vitality of land transfer has been increasing, but this market is still in its initial stage and there are large regional gaps. It has been reported that, at present, only 1% of family farms have more than 50 mu (1 mu = 666.67 m^2^ or 1 mu = 1/15 hectare) of farmland, and more than 80% of farmers have less than 10 mu [16]. Meanwhile, the proportion of land transfer in the Southeast coastal areas, such as Guangdong and Fujian, exceeds 50%, while this proportion in the Northwest and North China Plain provinces is less than 10% [17]. Therefore, it is worth considering why the proportion of land transfer is still so low in rural China.

Actually, academic communities have conducted relatively ample studies regarding the factors influencing farmland transfer [9,18,19]. First, there are external factors, which mainly include factors other than the farmers’ characteristics, such as land titling and land consolidation [15,17]. The second is internal factors, including household characteristics, such as householders’ age, education level and household assets [17,20,21]. Moreover, some scholars have argued that the unsophisticated public pension system is an important factor hindering the farmland transfer at the theoretical and empirical levels. The pension function provided by farmland is far greater than its productive function, so this pension function directly affects the land transfer behavior [17,22]. Unfortunately, the public pension system in rural China is too flawed to replace the function of farmland, which limits farmers’ enthusiasm participating in land transfer [22].

In 2009, the New Rural Pension System (NRPS) as a pilot was promoted in rural China. Some scholars have poured attention into the impact of the NRPS on land transfer. However, the studies have the following limitations. First, the existing studies are limited to a small region and the sample size is too small to represent the national characteristics, and it is difficult to control factors from regional differences [16]. Second, most of the data are from 2011 or earlier and cross-sectional, which makes it difficult to identify time heterogeneity and the endogeneity problem of the mutual causality between the NRPS and land transfer, leading to an overestimation in the effect of NRPS [23,24]. By 2017, the NRPS achieved full coverage in all county-level regions in China, thus it is necessary to ask whether or not the NRPS has formed a substitute of pension function from farmland for the elderly and whether or not it has promoted sustainability of land transfer.

The data used in this study were derived from the China Health and Retirement Longitudinal Study (CHARLS) from 2011 to 2015, which covered more than 20,000 households distributed in 450 villages across 150 prefecture-level cities. The panel tracking data can effectively reduce endogeneity problems and accurately quantify the impact of the NRPS on the land transfer. The remaining sections are arranged as follows. Section 2 displays the theoretical link between pension system and land transfer. Section 3 introduces in detail the data sources and empirical model. Section 4 gives the empirical results. Section 5 presents the heterogeneity analysis. Section 6 and Section 7 present discussion, conclusions and policy implications. Our findings will offer reference to improve the public social pension system for the elderly and enhance the vitality of the land rental market in rural China.

## 2. Theoretical Link between Pension System and Land Transfer

Currently, there are no institutional barriers to the transfer of labour from rural to cities for industrial development, but the major concern is the opportunity cost of non-agricultural employment for farmers, that is, the social pension function of cultivated land increases the opportunity cost of rural labour mobility, thus hindering the effective farmland transfer. Then, this study constructs a theoretical framework of the relationship between the new rural pension system and land transfer [24]. 

Assuming that the total labour endowment of each household is *L* and the area of contracted farmland is *T*. Farmers allocate their labour between *L_f_* in agricultural production and *L_w_* in the industrial sector. The income from agricultural production and migrant work are *F*(*L_f_*, *K*, *T_f_*) and *L_w_·W_t_*, respectively, where *K* is the material inputs, *T_f_* is the actual planting area for farmers, so the transferred land area is *T_t_ = T* − *T_f_ = T_t_*(*r*), that is, the transferred land *T_t_* is an increasing function of rent *r*. *F*(·) is a production function with constant return to scale, and the cross partial is positive. *W* is the expected wage for farmers, *W = W_a_* (*1 + c*) indicates that the expected non-agricultural wage for farmers should be equal to the current average wage (*W_a_*) and the present value of the social pension in the future (*cW_a_*). *W =* (*1 − r*) *F* (*L_f_*, *K*, *T_f_*) is satisfied under the opportunity cost trade-off between agriculture and non-agriculture. In practice, it is difficult to determine whether farmers can get a stable job due to the uncertainty of job opportunities, that is, *L = L_a_ + ϕ*, *ϕ* is impacted by jobs growth and its value can be negative or positive. In reality, the larger the rent (*r*) of farmland is, the more likely farmers tend to transfer out of land. 

Therefore, the benefits to farmers are as follows under the circumstances of perfect urban and rural labour market and competition between agricultural and non-agricultural sectors:

(1) $$R_{Max}=W_{t}\cdot L_{w}+rT_{t}$$

Constraints to maximize benefits:

(2) $$W_{t}=\left(1-r\right)\cdot F\left(L_{f},K,T_{f}\right)$$

(3) $$L_{f}+L_{w}=1$$

(4) ∅∈[−1, 1]

(5) $$0<L_{f},\;L_{w}\leq 1$$

The Lagrange function equation is constructed:

(6) $$M=W_{t}\cdot L_{w}+r\cdot T_{t}-\lambda \left[W_{t}-\left(1-r\right)\cdot F\left(L_{f},K,\;T_{f}\right)\right]$$

The equilibrium solution for farmers to obtain the maximum income:

(7) $$r=\frac{\left[W_{a}\left(1+c\right)\cdot \left(L_{w}+\phi \right)+F\left(L_{f},K,T_{f}\right)\right]}{F\left(L_{f},K,T_{f}\right)-\left[W_{a}\left(1+c\right)\right]\cdot \left(1-L_{w}-\phi \right)}$$

The larger *W_t_* means the bigger the *c* and the larger *r*, which indicates that the level of social pension coefficient determines the funds for land transfer, that is, the probability and area of land transfer. Once farmers’ opportunities to go out to work are damaged, cultivated land can still provide a kind of insurance in rural China due to the lack and imperfection of the social pension system for farmers. Therefore, when the coefficient of social pension is low, the farmers are most likely not to transfer out of farmland because the rent level is too low. For a long time, the social pension of rural residents in rural China is essentially land-centered informal security, and the security function of rural cultivated land is a rational response that farmers are forced to carry out self-security in the absence of social security. This long-term nature also determines that the forced promotion of rural land transfer may face greater risks when the social pension system is not perfect in rural China.

## 3. Materials and Methods

### 3.1. Data

The data used in this study were from the CHARLS for 2011, 2013 and 2015. The data involved over 20,000 families in 450 villages or communities across 150 prefecture-level cities in 30 provinces, municipalities and autonomous regions in China, and rural and urban samples accounted for 53% and 47%, respectively. To ensure the unbiases and representativeness of the sample, four stages of stratified sampling were adopted [17]. First, 150 prefecture-level municipalities were randomly selected from 30 provincial administrative units throughout the country (excluding the Tibet Autonomous region, the Hong Kong and Macao Special Administrative regions and Taiwan Province) based on the regional gross domestic product (GDP). Second, 450 villages or communities were randomly selected from 150 prefectural municipalities on the basis of the resident population per village or community in 2010. Third, 50–100 families were randomly selected in each village or community. Fourth, the personal information of all family members is included in principle, within which the householder and his spouse are the main responders. If the householder was not at home, a telephone interview is adopted and other family members provide supplementary answers.

The purpose of this study is to estimate the influence extent of NRPS on land transfer, thus the samples are limited to rural families. To prevent the results from being disturbed by data quality and other factors, this paper conducted a series of steps to clean the sample before the empirical analysis. First, the family samples and villages are combined to generate 6321 samples. Second, farm households without contracted farmland were excluded, representing a total of 56 households that accounted for 0.9% of the total samples. Third, farmers who rent in and rent out cultivated land were also excluded, which was a total of 273 households that accounted for 4.3% of the total samples, and the samples missing key indicators were removed, which was a total of 11 households. Finally, 5981 effective samples were obtained, accounting for 94.6% of the total samples.

**Figure 1:**
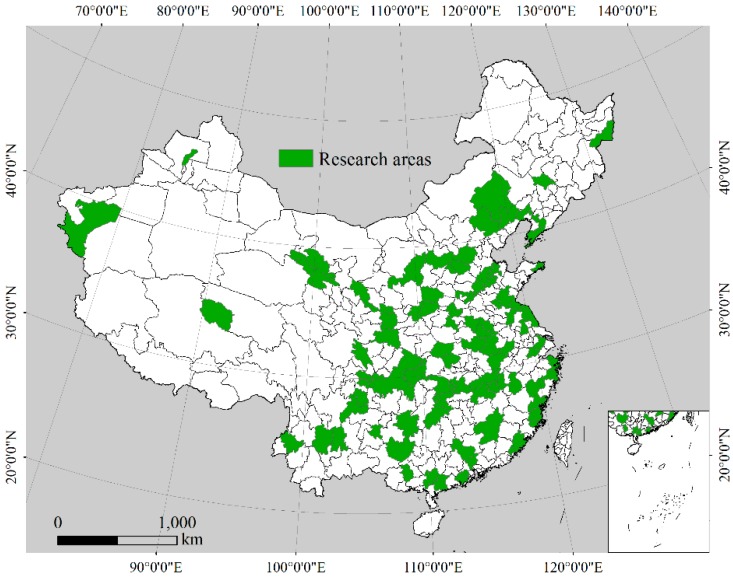
Distribution of study areas in China Health and Retirement Longitudinal Study.

The data used in this study consist of three phases. According to the status of the follow-up survey in the second and third phases, the number of samples fluctuates slightly in each year. The total sample number in 2011 was 5981 households, and there were 4899 and 5096 families in 2013 and 2015, and the corresponding tracking rate was 81.9% and 85.2%, respectively. The above samples were distributed across 236 villages and 101 prefecture-level cities (Figure 1). Furthermore, the data set includes three types of databases, such as the village database, the family database and the personal database. The village database includes the population, land use, the village economy and agricultural production in the village. The family database contains information on the family income and housing. The personal database contains the demographic information, educational history, work history and health history of the family members. It is worth mentioning that the information on land transfer, NRPS and other related indicators are included in this set of data, which makes this study carry out smoothly.

### 3.2. Statistical Description

**Figure 2:**
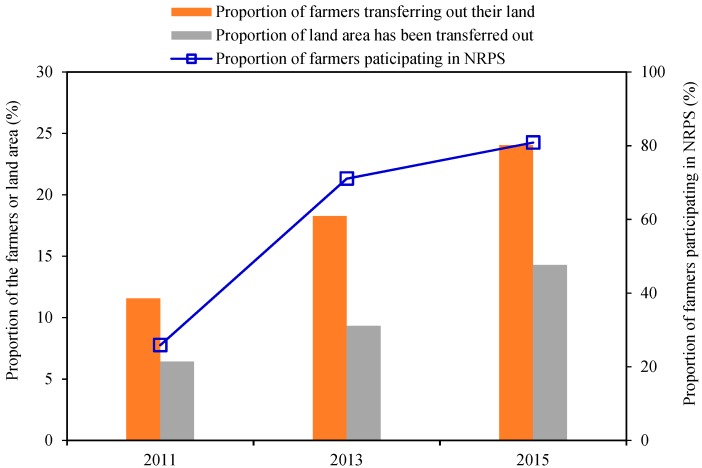
Proportion of farmers participating in NRPS and land transfer from 2011 to 2015.

Figure 2 shows the proportion of farmers participating in the NRPS, the proportion of farmers transferring out their land and the proportion of land area have been transferred in 2011, 2013 and 2015. First, from 2011 to 2015, the proportion of farmers participating in the NRPS increased from 25.87% at the beginning to 80.85% at the late stage. Second, the proportion of farmers transferring out their land increased from 11.56% at the initial stage to 24.04% at the end of the study period, which represents a 13% increase. Third, the proportion of farmland transferred out increases from 6.43% to 14.26% in the late stage, representing a total increase of nearly 8%. In summary, the participation rate in NRPS and farmland transfer all showed an upward trend during the study period. 

Table 1 and Table 2 show a comparison of the ratio of land transferred each year and the ratio of land area transferred under the two scenarios of participating and not participating in the NRPS. First, examining the ratio of farmers who transferred out land, the ratio reaches 21.5% when farmers participate in the NRPS, whereas the ratio is only 9.6% when farmers do not participate in the NRPS. In each year, the ratio mentioned above is more than 20% if farmers participate in the NRPS; otherwise, it is less than 17% for all years.

Second, concerning the land area has been transferred out, the average scale of land transferred when farmers participate in the NRPS is 0.652 mu, whereas it is 0.378 mu for non-participating farmers, which is 58% of the former. There are significant differences in each year. In 2011 and 2013, there were significant differences in land area transferred between different groups, namely farmers who participated in NRPS and did not participate in NRPS, and the land area transferred for the former was greater than that of the latter. However, there was no significant difference between the two groups in 2015. Initially, the statistical results seem to show that participation in the NRPS can increase the probability of farmers participating in land transfer and the scale of land transfer. However, to determine whether the difference in the proportion and scale of rural households transferring land in the two groups is caused by participating in the NRPS and to identify the effect of participation in the NRPS, we still need to carry out a strict econometric test.

### 3.3. Empirical Model Specification

This study focuses on whether farmers’ participation in the NRPS promotes farmland transfer; it measures the level of land transfer by the probability of transferring out of the land and the scale of the transferred land. In the CHARLS questionnaire, the land that farmers transferred in is not only from other households but also from village collectives, so it cannot reflect the behavior of land transfer among individuals and cannot fully reflect the development level of the land rental market. In contrast, the land transferred out by farmers mainly flows to other farmers, large planters and agricultural enterprises, so the probability and the scale of land transferred can be weakened by the influence of village collectives and can reflect the development level of the land rental market. The empirical estimation strategies used in this study are as follows.

#### 3.3.1. Panel Logit Model

If the farmers transfer out their land, the dependent variable is assigned a 1 and otherwise 0. Considering that the values of the dependent variables are 0 or 1, the panel logit model is adopted to estimate them [25,26,27]. The model setting is as follows:

(8) $$P_{it}=E\left(Y=1|Y_{it}\right)=\frac{1}{1+e^{-Y_{it}}}$$

(9) $$Y_{it}=\alpha_{0}+\alpha_{1}\cdot NRPS_{it}+\alpha_{ik}\cdot \sum X_{itk}+\mu_{it}$$

where *P_it_* indicates the probability of the *i*th farmer transferring out his or her land in the *t*th year; *E*(*Y = 1|Y_it_*), which represents the probability of a farmer transferring out land given a value of *Y_it_*; *NRPS_it_* is the core independent variable and indicates whether the *i*th farmer participates in the NRPS in the *i*th year, in which case, it is 1, and otherwise is 0. *X_itk_* indicates a range of other control variables (*k* = 4), which include variables of householder’ characteristics, family, village and year. In particular, the householder characteristics include householder’s age, education level and health status; and family characteristics include whether to participate in the new rural cooperative medical system, farmland resource endowment, dependency ratio, productive assets and non-agricultural income. Village features include geographical location, infrastructure, ratio of migrant population, unemployment insurance and land rent. Moreover, dummy variables for cities and years are included to control other unobservable factors, such as culture and climate. The definition and statistical description of each variable are shown in Table 3. *α*_0_, *α*_1_ are all parameters to be estimated.

#### 3.3.2. Panel Tobit Model

Considering that the scale of transferred farmland is positive, some farmers do not participate in the rural land rental market and the scale of farmland transferred out is zero; the dependent variable is the left truncated data, with zero as the minimum value. To estimate this class of dependent variables, the panel tobit model is used [25,28,29]. The model settings are as follows:

(10) $$y_{it}=\beta_{0}+\beta_{1}\cdot NRPS_{it}+\beta_{ik}\cdot \sum X_{itk}+\gamma_{it}$$

(11) $$y_{it}=\{\begin{array}{c} y_{it}^{*},\;y_{it}^{*}>0\\ 0,\;\;\;\;y_{it}^{*}\leq 0 \end{array}$$

where *y_it_* and $y_{it}^{*}$ represent the scale of the *i*th farmer transferring out farmland in the *t*th year. When the scale of farmland transfer is greater than 0, the value of the land scale is assigned to the dependent variable, while, when the scale of transferred farmland is equal to zero or farmers do not participate in land transfer, the dependent variable is assigned the value zero. *NRPS_it_* and *X_itk_* have the same meaning as in Equation (1). *β*_0_, *β*_1_ and so on are all parameters to be estimated.

#### 3.3.3. DID Model

To test the robustness of the results, we further use the panel data of 2008 and 2011 to construct DID (Difference in Difference) model to identify the changes of land transfer behavior before and after the new rural pension system pilot. In 2008, only the data of Zhejiang Province and Gansu Province were included, and the new rural pension system has not yet been piloted, while the selected survey in 2011 has been piloted in the two provinces above. The DID model was used to identify the changes of land transfer behavior before and after farmers’ participation in the system, and other control variables are further introduced into the econometric model to test the statistical significance of this effect. DID model is shown as follows [30]:

(12) $$Y_{it}=\beta_{0}+\beta_{1}T+\beta_{2}D_{i}+\beta_{3}D_{i}T+\beta_{4}X_{it}+\varepsilon_{it}$$

where *Y_it_* represents the probability of transferring land and the area of transferred land, *T* is a time dummy, *D_i_* is a variable of whether to be insured or not, *D_i_* × *T* is the net effect of new rural pension system on farmers’ land transfer behavior, and *ɛ_it_* is an unobservable error term; *β*_0_, *β*_1_, *β*_2_, *β*_4_ are the parameters to be estimated.

### 3.4. Endogeneity Problem Analysis

The NRPS began to pilot in 2009, whether a village is included in the pilot is strictly exogenous for a farmer, but the village may not have been randomly selected. Statistics show that, prior to 2011, a total of 107 villages in CHARLS were included in the pilot, accounting for 45% of the total villages. One possible assumption is that villages with a higher proportion of land transfer were more likely to be selected for pilot because the government would find it appealing to include these villages first in the new rural social pension insurance program. If there is an endogeneity problem, as discussed above, then there must be endogeneity problems of mutual causation between participating in the NRPS and land transfer, and the estimated coefficient of the NRPS is biased [18,31].

This study needs to conduct a regression test of whether a village is included in the NRPS pilot to judge whether there are significant differences in the characteristics of the included villages. The decision model for whether a village should be included in the NRPS pilot is as follows:

(13) $$NRPS_{jt}=\phi +\lambda_{k}\cdot \sum X_{jtk}+\rho_{jt}$$

where NRPSjt indicates the dependent variable representing whether the jth village is included in NRPS in the tth year. If it is included, NRPSjt = 1; otherwise, it is 0. Xjtk indicates a range of factors that affect the inclusion of the village in the NRPS, such as village welfare, ratio of elderly population, geographical location, infrastructure, ratio of migrant population and economic development level. The remaining parameters are to be estimated. Table 3 shows the results of the logit and probit models, which shows that none of the variables at the village level affect whether the village is included in the NRPS, and the results of the two models are highly consistent; that is, whether a village is included in the NRPS is random, and there is no endogeneity problem in the study.

Table 4 presents a statistical description of the variables. First, examining farmers’ participating in the land rental market, the proportion of land transfer increased from 12% in the initial stage to 24% in the late year. The scale of transferred land grew from 0.34 mu to 0.82 mu per household, with an increase of 142%. Second, considering farmers’ participation in the NRPS and a new rural cooperative medical system, the participation rate of both continued rising during the study period; the participation rate in NRPS increased from 26% in the early stage to 81% in the late stage and the participation rate in the new rural cooperative medical system increased from 82% to 92%. Third, turning to family characteristics, the householders’ average age is over 63, and the dependency ratio is increasing, from 0.75 at the beginning to 0.81 at the end, which means that each member of the effective labour force needs to support 0.81 non-workers each year; that is, the proportion of the ageing people in rural China has grown and the pressure on family support is increasing. Finally, at the village level, 20% of the villages have carried out land levelling and 64% of the villages had access to cement roads. The number of rural migrant workers increased from 30% at the beginning to 61% at the end. In addition, the average rent of transferred farmland per mu is approximately 330 yuan.

## 4. Results

### 4.1. The Impact of the NRPS on the Probability of Land Transfer

Before the model fitting, the variance inflation factor (VIF) was used to test the collinearity of the independent variables. The results show that the VIF value of all variables is less than 1.76, and the average VIF value is 1.2, indicating that there is no serious collinearity problem between variables. Theoretical inference indicates that participation in the NRPS can have a positive impact on farmland transfer. Table 5 shows the influence of participating in the NRPS on land transfer, and the results support that participating in the NRPS has significantly improved the land transfer rate.

Specifically, model 1 only included whether farmers participated in the NRPS as an independent variable. The results showed that the NRPS was significantly positive at the 1% significance level, and the marginal effect was 0.142. That is, participation in the NRPS can increase the land transfer rate by 14.2%. On the basis of model 1, model 2 took into account the characteristics of the new rural cooperative medical system and the householder. The coefficient of the NRPS was still significant at the 1% significance level, and the marginal effect was 0.133. On the basis of model 2, model 3 continued to incorporate family characteristics variables; the significance of the NRPS coefficient did not change, but the coefficient decreased to 0.836, and the marginal effect decreased to 0.128. On the basis of model 3, model 4 continued to incorporate village characteristics and region and year dummy variables. It is worth noting that the significance of the NRPS coefficient remained unchanged; at this time, the coefficient was 0.801, and the marginal effect dropped to 0.127. The results of model 4 showed that, all else being equal, participation in the NRPS can significantly increase the land transfer rate of farmers by 12.7%.

The results of control variables are basically aligned with most study’s conclusions [16,18]. Taking model 4 as an example, participating in the new rural cooperative medical system did not effectively promote land transfer. There is a U-shaped relationship between householders’ age and the probability of land transfer, in which the inflection point is 62 years old, that is, when the householder is younger than 62 years old, he or she tends to transfer in farmland, while, when the householder is older than 62 years old, he or she tends to transfer out their farmland. It may be that older householders can no longer engage in agricultural production and are willing to transfer their farmland [15]. The coefficient of the dependency ratio was significantly positive at the 1% level of significance, which indicates that, when the dependency ratio increases, farmers become more inclined to transfer out farmland. The reason may be that a higher dependency ratio indicates greater pressure from the need to provide family support, and farmers may find it impossible to meet the basic living expenses by relying on agriculture. Therefore, these farmers tend to transfer out farmland and engage in non-agricultural employment [17]. In addition, good infrastructure, a higher rate of migrant workers, a better level of economic development and higher rent at the village level can increase the proportion of land transfer to a certain extent [15].

### 4.2. The Impact of the NRPS on the Land Area Transferred

To test the robustness of the model, four additional models are used to fit the model. Model 1 only included the new rural pension system, and the results showed that NRPS was significantly positive at the significance level of 1%, and its marginal effect was 0.128; that is, participating in the NRPS increased the scale of farmers transferring out of farmland by 12.8% compared with the farmers who did not participate in the NRPS. On the basis of model 1, models 2 and 3 continued to incorporate householder and family characteristics, respectively. The results showed that the coefficient of NRPS remained significantly positive at the significance level of 1%. On the basis of model 3, model 4 continued to incorporate village characteristics, prefecture-level cities, and the year dummy variable, and the final results showed that participating in the NRPS continues to significantly increase the land area. The results showed that, under the same conditions, farmers who participate in the NRPS increase the scale of farmland transfer by 11.2%.

The fitting results of the control variables are similar to those in Table 5. First, families with higher dependency ratios tend to transfer out farmland to engage in non-agricultural employment. Second, in villages with good infrastructure, more migrant workers, higher levels of economic development and higher rent for land, the scale of farmers transferring out of farmland is generally higher. As shown in Table 5 and Table 6, participation in the NRPS has indeed improved the efficiency of the land rental market, with other conditions being held the same: the farmland transfer rate increased by 12.7% and the farmland area transferred out increased by 11.2%.

### 4.3. Robustness Test of Empirical Results

Table 7 reports the robustness test based on a DID model. There is no doubt that the result of DID estimation is very consistent with the conclusion above. We are concerned about the DID estimators, that is, the coefficients of the interaction terms between time variable and NRPS are positive and have passed the significance test at the level of 5%, indicating that the pension system effectively promotes the farmers to transfer out of farmland. The mechanism of the impact of pension system on land transfer is that cultivated land is not only an important means of production for farmers, but also an important pension security for farmers. To a certain extent, the pension system weakens the function of cultivated land to bear the function of pension security, and reduces the dependence of insured farmers on family cultivated land. Therefore, farmers who participate in the insurance are more willing to transfer land and increase the scale of cultivated land has been transferred out.

## 5. Heterogeneity on the Impact of NRPS on Land Transfer

First, the NRPS has been gradually promoted since 2009, different farmers have participated in the NRPS at different times; does different participation time have different influences on land transfer? Second, the insured standard of the NRPS is divided into eight classes: 100, 200, 300, 400, 500, 600, 800 and 1000 yuan per year. Different insured standards are expected to result in differences in the future pension, so, with an increase in the insured standard, do farmers become more willing to transfer out their farmland? Third, it was found that some families participate not only in the NRPS but also in commercial pension insurance, while some families only participate in the NRPS. Is there a significant difference in the effect of the NRPS on the two types of families? In the next section, the samples need to be grouped for analysis to test the three questions above.

### 5.1. Heterogeneity of Participation Time in NRPS

The total samples are divided into two groups according to participation time in the NRPS, that is, fewer than five years and more than five years. Table 8 presents the time heterogeneity test of the NRPS. When the dependent variable is whether the farmland is transferred, the coefficient of the NRPS is significantly positive at the 1% significance level in both groups. The marginal effects were 0.042 and 0.203 in the samples with fewer than five years and more than five years, respectively, participation in the NRPS increased farmland transfer rate by 4.2% and 20.3%, respectively, and the latter was nearly five times that of the former. When the dependent variable is the scale of transferred farmland, the coefficients of the NRPS in the groups of samples are also significantly positive at the 1% significance level, and the marginal effects of the two groups are 0.031 and 0.173, respectively. That is, participation in the NRPS increases the area of farmland transferred by 3.1% and 17.3%, and the latter is 5.6 times that of the former. Therefore, the longer the farmer has participated in the NRPS, the more it improves farmers’ enthusiasm for participating in land transfer.

### 5.2. Heterogeneity of the Insured Standards in NRPS

The samples were divided into two groups according to the differences in the insured standards of the NRPS. In the first group, the insured amount per capita is less than 500 yuan per year, including the 100, 200, 300 and 400-yuan grades, and the second group includes the per capita payments of more than 500 yuan per year, including the 500, 600, 800 and 1000-yuan grades. Table 9 presents a heterogeneity test of the effect of the NRPS based on different insured standards. When the dependent variable is whether to transfer out farmland, if the insured standard is more than 500 yuan per year, the marginal effect of the NRPS is 0.152; that is, participation in the NRPS increases the probability of transferring out farmland by 15.2% for this group. Meanwhile, if the insured amount is less than 500 yuan per year, the marginal effect is 0.58. When the dependent variable is farmland area transferred out, the higher the standard of payment, the greater the transferred farmland area will be. The marginal effects of the higher and lower groups are 0.161 and 0.035, and the difference between them is 4.6 times.

### 5.3. Heterogeneity of Participation in Commercial Pension Insurance

The total samples are divided into farmers who only participate in NRPS and farmers who participate in both NRPS and commercial insurance. Table 10 shows the effect of the NRPS on the two groups of samples. Whether the dependent variable is the probability of transferring land or the area of transferred land, participation in the NRPS has a strong promotion effect for farmers who have not participated in commercial insurance, resulting in an increase of 43% and 129%, respectively.

## 6. Discussion

The implementation of the new rural pension system has lots of impact on the rural system, among which the pension system has the most obvious impact on land transfer. Since the 1980s, cultivated land is the lifeblood for farmers has been widely popular in political and academic circles. That is to say, cultivated land is not only a means of production for farmers, but also as a kind of old-age security. However, there have been large-scale rent-free land transfer and land abandonment in rural China in recent years, which indicates that the function of cultivated land as a means of production or old-age security is declining [13,16,32]. According to the actual investigation, the pension function of cultivated land is far from meeting the basic consumption demand of the elderly.

**Figure 3:**
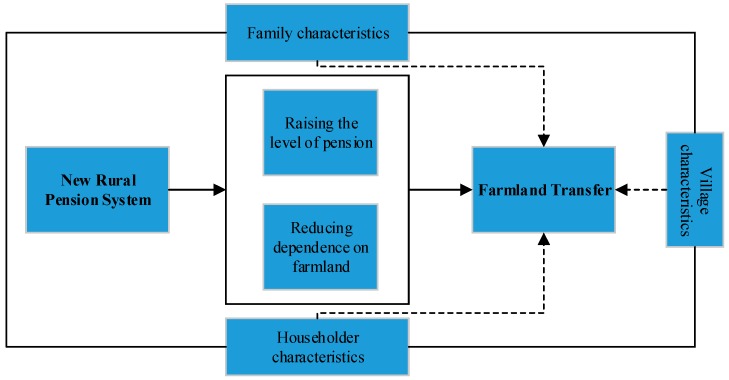
Mechanism analysis of the impact of NRPS on land transfer.

Meanwhile, to improve the level of rural pension security, the government began to pilot a new rural pension system in China in 2009, and all rural residents can enjoy the new rural pension. Theoretical inference shows that the implementation of the system is conducive to promoting the farmland transfer, and the empirical results of this study also show that the system does improve the probability and area of farmers transferring to land. Figure 3 shows that its mechanism. The pension system to some extent improves the level of expected old-age security, reduces the excessive dependence of farmers on farmland for the aged and liberates farmers from cultivated land, and thus improves the vitality of land transfer market in rural China.

With the prolongation of population life expectancy and low birth rate brought by family planning policy, the problem of rural population aging is prominent because a large number of young and middle-aged labour forces migrate to cities. The proportion of the population aged 65 or above in rural China as high as 14% of the total population, 2.8% and 2.6% higher than that of cities and towns, respectively. In the context of an aging population in rural areas, to realize the orderly transfer of farmland management rights and develop moderate scale operation, we must perfect the public social pension system in rural China, which is the realistic way to effectively promote the sustainability of farmland transfer. Farmland transfer is facing great constraints and difficulties in rural China, and the most important problem is how to separate the pension function and the production function of farmland. Therefore, the task at this stage is to gradually improve the public social pension system in rural areas and improve the treatment level of the elderly, so as to weaken the pension function of farmland and reduce the worries about the farmland transfer. In addition, the NRPS covers more than 80% of the rural population, and it would be difficult to further improve its coverage. However, the proportion of farmland has been transferred in rural China is still less than 30%, and the land rental market is still at an elementary level. The government’s next efforts will still be directed toward effectively improving the level of farmland transfer. 

However, the expansion of the farmland scale may lead to potential negative issues, such as environmental pollution. Evidence from Europe suggests that the larger farm has a negative effect on air and water resources [33]. A study from the mountainous areas of southwest China also believes that farmland is affected by excessive application of machinery and fertilizer with the expansion of farm scale [34]. However, so far, production costs and benefits have been optimized with the expansion of farmland in China.

It is worth noting that this study may have the following limitations. First, the householder’s age in these data set is generally over 45 years old, and the samples may have some bias, but most studies show that the age of householder’s age in rural China exceeds 50 years old, so this data set can still better reflect the family characteristics in rural China. Second, to overcome the two-way causal relationship between NRPS and land transfer, this study first identified that the implementation of NRPS is mandatory by the government and there is no strong selectivity problem. Meanwhile, robust tests such as panel data model and DID model are also adopted in this study and the results showed that NRPS is the cause and land transfer is the result. In addition, the infulence extent of NRPS on land transfer varies significantly with different farmers, such as different insurance time and insurance quota. The heterogeneity analysis mentioned above is essentially to identify the response of different groups to the pension system by grouping regression to improve the relevant policies for policymakers. Finally, from a welfare perspective, existing studies show that land transfer does improve household income and labour productivity for farmers [7,9], but these studies are still insufficient, especially the follow-up investigations on the real performance of the transferred land. Therefore, more details on the land transfer activities should be concerned in the future.

## 7. Conclusions and Policy Implications

The impact of new rural pension system on farmland transfer under the background of aging has received much attention in various research fields. However, there are so many limitations in these studies that it is impossible to accurately identify the influence extent of the NRPS. To fill this gap, we developed a panel logit model and panel tobit model to systematically evaluate the impact of the NRPS on farmland transfer in rural China. The main findings of this study are as follows. First, the participation rate in the NRPS and the proportion of farmland transfer both continuously increased, reaching 80.85% and 24.04% in 2015, respectively. Second, the NRPS has significantly improved the operational efficiency of the farmland rental market. Under the same conditions, the proportion of farmers who transferred out farmland increased by approximately 13%, and the farmland area has been transferred out increased by 11.2% due to participating in the NRPS. Finally, considering the difference in farmers’ participation time and insured standards, the effect of the NRPS also exhibits great heterogeneity. As an example, the proportion and scale of farmers who transferred out of farmland show a significant upward trend with an increase in the participation time and the insured standard. In addition, the establishment of the NRPS has a stronger promoting effect on the probability and scale of farmland transfer in rural China for the farmers who do not participate in other commercial pension insurance.

The conclusions of this study has some policy implications. First, the current proportion of farmers buying commercial pension insurance is only 4.2%, and more than 95% of farmers still need to rely on the NRPS as a means of old-age security. Therefore, the government should continue to promote the coverage of NRPS, especially among those farmers who do not participate in other commercial pension insurance programmes. Second, although the NRPS basically covered all of China by the end of 2012 and the number of farmers participating in the NRPS has increased annually, the insured amount is not high. Statistics show that the insured amount per capita is approximately 204 yuan per year, and it is expected that the monthly pension will drop to less than 100 yuan in the future, which is far lower than that of commercial pension insurance or urban residents’ pension insurance. The government needs to encourage farmers to raise the insured standard of the NRPS so that they can obtain higher pension benefits and reduce their dependence on farmland. Third, the local government should speed up the establishment of a rural land transfer information platform, clear the information communication channels between the supply and the demand for farmland and reduce the transaction costs of land transfer. These actions will help to improve the efficiency of land transfer and realize the optimal allocation of farmland resources.

## Figures and Tables

**Table 1:** Ratio of farmers who transferred land by participating and non-participating in NRPS.

Year	Participating in NRPS		Non-Participating in NRPS		Difference	*t*-Value
	Mean	S. D	Mean	S. D		
2011	0.221	0.011	0.079	0.004	0.14 ***	15.37
2013	0.217	0.007	0.097	0.008	0.12 ***	9.97
2015	0.208	0.006	0.164	0.011	0.05 ***	3.34
Total sample	0.215	0.004	0.096	0.004	0.12 ***	20.29

Note: *, **, *** are significantly different from zero at the 10%, 5% and 1% levels, and the same below.

**Table 2:** Land area transferred out by farmers participating and not participating in the NRPS.

Year	Participating in NRPS		Non-Participating in NRPS		Difference	*t*-Value
	Mean	SD	Mean	SD		
2011	0.679	0.102	0.242	0.019	0.44 ***	6.33
2013	0.496	0.043	0.328	0.042	0.17 **	2.31
2015	0.798	0.057	0.971	0.172	−0.17	−1.37
Total sample	0.652	0.035	0.378	0.032	0.274 ***	5.63

**Table 3:** Determinants of the NRPS at the village level.

Variables	Logit	Probit
Does your village have the old rural pension system (yes = 1, otherwise = 0)	0.479	0.289
	(1.03)	(1.12)
Does your village issue pension to people older than 65 (yes = 1, otherwise = 0)	0.767	0.459
	(1.64)	(1.61)
Does your village have a minimum living allowance (yes = 1, otherwise = 0)	0.403	0.207
	(1.05)	(0.92)
Ratio of people older than 65 (%)	−1.689	−0.908
	(−1.01)	(−0.99)
Village has implemented land titling in the past 5 years (yes = 1, otherwise = 0)	−0.318	−0.160
	(−0.81)	(−0.71)
Village has implemented land consolidation in last decade (yes = 1, otherwise = 0)	0.343	0.128
	(0.64)	(0.44)
Village is located in plains (yes = 1, otherwise = 0)	−0.655	−0.406
	(−1.42)	(−1.55)
Does your village have paved roads (yes = 1, otherwise = 0)	0.651 *	0.388 *
	(1.77)	(1.81)
Proportion of emigration in village (%)	−0.280	−0.196
	(−0.38)	(−0.45)
Village has experienced serious disasters in last decade (yes = 1, otherwise = 0)	−0.061	−0.010
	(−0.18)	(−0.05)
Net income per capita in village (yuan)	0.330	0.211 *
	(1.55)	(1.80)
Regional dummies	Yes	Yes
Constant	−2.367	−1.491
	(−1.29)	(−1.43)
Pseudo R^2^	0.216	0.214
Log likelihood	−127.42	−127.80
Number of observations	236	236

Note: Standard errors are adjusted for clusters in the village; * is significantly different from zero at the 10% level.

**Table 4:** Definitions and statistical descriptions of variables.

Variables	Definitions	2008 (*n* = 1213)		2011 (*n* = 5981)		2015 (*n* = 5096)	
		Mean	SD	Mean	SD	Mean	SD
Ratio of farmer who rented out land	Rented out farmland in last year (yes = 1, no = 0)	0.09	0.23	0.12	0.32	0.24	0.40
Farmland area rented out	Amount of farmland rented out in last year (mu per farm household)	0.31	2.12	0.36	2.34	0.82	4.19
**Independent variable**							
NRPS	Has participated in new rural pension system (yes = 1, no = 0)	0.14	0.25	0.26	0.44	0.81	0.42
**Householder characteristics**							
Head’s age	Householder’s age (years old)	57.34	9.64	63.9	10.31	66.11	10.15
Head’s education level	Has completed middle school (yes = 1, no = 0)	0.13	0.19	0.25	0.23	0.32	0.30
Head’s health status	Self-assessment physical health (poor = 1, good = 2, excellent = 3)	1.67	0.45	1.87	0.50	1.93	0.50
**Family characteristics**							
Farmland size in family	Amount of cultivated land per farm household (mu)	6.76	11.34	6.35	10.09	5.74	9.43
Land scale per capita	Farmland size per capita in farm household (mu)	1.02	1.32	0.92	1.26	0.69	1.21
Dependency ratio	Number of dependants divided by the number in the labour force	0.71	0.29	0.75	0.26	0.81	0.37
Productive assets	Total value of family productive assets (yuan)	1239.23	3023.12	1022.78	4236.18	987.72	2921.93
Land contract rights certificate	Has contract certificate for land (yes = 1, no = 0)	0.32	0.54	0.29	0.46	0.38	0.45
New rural cooperative medical system	Has participated in new rural cooperative medical system (yes = 1, no = 0)	0.43	0.12	0.82	0.27	0.92	0.22
**Village characteristics**							
Land reallocation	Land reallocation in last decade (yes = 1, no = 0)	0.15	0.37	0.18	0.38	0.21	0.38
Located in plains	Located in plains (yes = 1, no = 0)	0.43	0.47	0.32	0.47	0.33	0.47
Cement roads	Village has a cement road (yes = 1, no = 0)	0.52	0.50	0.57	0.50	0.64	0.50
Ratio of emigration	Ratio of population outflow	0.30	0.41	0.30	0.27	0.61	0.46
Natural disasters	Has experienced serious disasters in the past five years (yes = 1, no = 0)	0.39	0.32	0.44	0.50	0.43	0.5
Income per capita	Net income per capita in village (yuan)	3428.28	4000.41	3552.79	3000.75	3467.97	2809.50
Farmland rent	Average rent per mu in village (yuan/mu)	310.32	890.45	333.48	908.32	335.56	900.37

**Table 5:** Impact of NRPS on the probability of transferring farmland.

Variables	Model 1	Model 2	Model 3	Model 4
NRPS	0.929 *** [0.142]	0.873 *** [0.133]	0.836 *** [0.128]	0.801 *** [0.127]
	(20.30)	(17.40)	(12.10)	(10.45)
Head’s age		−0.097 ***	−0.049	−0.062 **
		(−4.10)	(−1.60)	(−1.96)
Head’s age ^2		0.001 ***	0.0005	0.0005 *
		(3.58)	(1.30)	(1.67)
Head’s education level		0.132 ***	0.143**	0.287
		(2.71)	(2.51)	(1.12)
New rural cooperative medical system		−0.103 **	−0.089	0.061
		(−2.05)	(−1.45)	(0.76)
Dependency ratio			0.379 ***	0.279 ***
			(5.74)	(3.51)
Productive assets			−0.030 ***	−0.024 ***
			(−3.97)	(−2.96)
Land contract rights certificate			0.207 ***	0.090
			(3.32)	(1.20)
Ratio of emigration				0.442 ***
				(3.42)
Natural disasters				−0.170 ***
				(−2.61)
Log (Income per capita in village)				0.237 ***
				(5.43)
Log (Farmland rent in village)				0.072 ***
				(5.62)
Regional dummies	No	No	No	Yes
Year dummies	No	No	No	Yes
Constant	−2.216 ***	1.326 *	−0.460	−2.070 *
	(−52.74)	(1.71)	(−0.44)	(−1.85)
Wald chi2(1)	412.29	450.35	340.62	614.06
Number of observations	15,976	15,830	13,295	13,295

Note: (1) * ** *** indicate significantly different from zero at the 10%, 5% and 1% levels. (2) [] represents the marginal effect, () represents the *t*-value. (3) The standard error was adjusted for clusters in the villages; (4) Other variables such as land reallocation were controlled in model 4. These variables were not significant, and the results were not significantly changed when the above variables were removed. Therefore, the results are not reported in Table 5. (5) Regional dummies and year dummies were not reported due to space limitations, and the same below.

**Table 6:** The impact of the NRPS on the scale of farmland area transferred out.

Variables	Model 1	Model 2	Model 3	Model 4
NRPS	0.098 *** [0.128]	0.085 *** [0.115]	0.089 *** [0.119]	0.082 *** [0.112]
	(13.39)	(10.28)	(7.65)	(4.33)
Head’s age		−0.014 ***	−0.005	−0.008
		(−3.06)	(−0.98)	(−1.56)
Head’s age ^2		0.001 ***	0.001	0.001
		(2.68)	(0.92)	(1.48)
Head’s education level		−0.019 **	−0.036 ***	−0.034
		(−2.47)	(−3.64)	(−0.71)
New rural cooperative medical system		−0.062 ***	−0.045 ***	0.001
		(−6.43)	(−3.89)	(0.03)
Dependency ratio			0.068 ***	0.030 **
			(5.54)	(2.28)
Productive assets			−0.006 ***	−0.007 ***
			(−4.81)	(−4.83)
Land contract rights certificate			0.057 ***	0.014
			(4.57)	(1.06)
Ratio of emigration				0.087 ***
				(4.02)
Natural disasters				−0.041 ***
				(−4.11)
Log (Income per capita in village)				0.025 ***
				(3.58)
Log (Farmland rent in village)				0.010 ***
				(5.84)
Regional dummies	No	No	No	Yes
Year dummies	No	No	No	Yes
Constant	0.127 ***	0.712 ***	0.302 *	0.165
	(23.47)	(4.60)	(1.70)	(0.91)
Wald chi2	179.42	291.11	229.89	488.78
Number of observations	15,976	15,976	13,295	13,295

Note: *, **, *** are significantly different from zero at the 10%, 5% and 1% levels, respectively.

**Table 7:** Robustness test of the results of DID estimation.

Dependent Variables	Probability of Transferring Farmland	Farmland Area Transferred Out
Interaction terms	0.487 **	0.243 **
	(2.32)	2.12
Time effect	−0.003	−0.23
	(−0.03)	(−0.45)
Group effect	−0.524 ***	−0.343 **
	(−2.87)	(−2.13)
Other variables	Yes	Yes
Regional dummies	Yes	Yes
Chibar2	134.23	231.43
Number of observations	2315	2315

Note: **, *** are significantly different from zero at the 5% and 1% levels, respectively.

**Table 8:** Heterogeneity test of participation time (PT) for the NRPS.

Variables	Dependent Variable: Leaser = 1, Otherwise = 0		Dependent Variable: Log (Farmland Area)	
	PT <5 years	PT ≥5 years	PT <5 years	PT ≥5 years
NRPS	0.423 *** [0.042]	2.211 *** [0.203]	0.031 *** [0.031]	0.173 *** [0.173]
	(7.34)	(18.32)	(3.52)	(6.41)
Constant	−1.228	−1.979	0.194	0.069
	(−1.05)	(−1.06)	(1.06)	(0.30)
Wald chi2	505.31	645.93	479.32	307.97
Number of observations	12,819	6329	12,819	6329

Note: *, **, *** are significantly different from zero at the 10%, 5% and 1% levels, respectively. [] represent the marginal effect of the NRPS and () represent the *t*-value. The standard error was adjusted for clusters in the village and other variables are not reported due to space constraints.

**Table 9:** Heterogeneity test of the insured standards (IS) of the NRPS.

Variables	Dependent Variable: Leaser = 1, Otherwise = 0		Dependent Variable: Log (Farmland Area)	
	IS <500 yuan	IS ≥500 yuan	IS <500 yuan	IS ≥500 yuan
NRPS	0.524 *** [0.058]	1.500 *** [0.152]	0.035 *** [0.035]	0.161 *** [0.161]
	(8.42)	(14.73)	(3.30)	(6.29)
Constant	−0.322	1.558	0.297^*^	0.358^*^
	(−0.29)	(0.97)	(1.68)	(1.68)
Wald chi2	455.05	498.94	409.43	327.37
Number of observations	12,497	6608	12,497	6635

Note: *, **, *** are significantly different from zero at the 10%, 5% and 1% levels, respectively.

**Table 10:** Heterogeneity test for participating in commercial pension insurance.

Variables	Dependent Variable: Leaser = 1, Otherwise = 0		Dependent Variable: Log (Farmland Area)	
	Without Commercial Insurance	With Commercial Insurance	Without Commercial Insurance	With Commercial Insurance
NRPS	0.575 *** [0.072]	0.573 *** [0.063]	0.068 *** [0.078]	0.035 *** [0.035]
	(3.95)	(9.00)	(2.73)	(3.48)
Constant	4.360 **	−0.946	1.099 ***	0.201
	(2.31)	(−0.77)	(3.03)	(1.07)
Wald chi2	239.56	485.89	287.39	329.32
Number of observations	3253	10,120	3253	10,120

Note: *, **, *** are significantly different from zero at the 10%, 5% and 1% levels, respectively.
